# A Novel Prognostic Model of Endometrial Carcinoma Based on Clinical Variables and Oncogenomic Gene Signature

**DOI:** 10.3389/fmolb.2020.587822

**Published:** 2021-01-07

**Authors:** Fang Deng, Jing Mu, Chiwen Qu, Fang Yang, Xing Liu, Xiaomin Zeng, Xiaoning Peng

**Affiliations:** ^1^Department of Epidemiology and Health Statistics, Xiangya School of Public Health, Central South University, Changsha, China; ^2^School of Mathematics and Statistics, Hunan Normal University, Changsha, China; ^3^Department of Pathology and Pathophysiology, Hunan Normal University School of Medicine, Changsha, China; ^4^Department of Pathophysiology, Jishou University School of Medicine, Jishou, China

**Keywords:** endometrial carcinoma, cancer genomics, the Cancer Genome Atlas (TCGA), integrative model, prognosis

## Abstract

Due to the difficulty in predicting the prognosis of endometrial carcinoma (EC) patients by clinical variables alone, this study aims to build a new EC prognosis model integrating clinical and molecular information, so as to improve the accuracy of predicting the prognosis of EC. The clinical and gene expression data of 496 EC patients in the TCGA database were used to establish and validate this model. General Cox regression was applied to analyze clinical variables and RNAs. Elastic net-penalized Cox proportional hazard regression was employed to select the best EC prognosis-related RNAs, and ridge regression was used to construct the EC prognostic model. The predictive ability of the prognostic model was evaluated by the Kaplan–Meier curve and the area under the receiver operating characteristic curve (AUC-ROC). A clinical-RNA prognostic model integrating two clinical variables and 28 RNAs was established. The 5-year AUC of the clinical-RNA prognostic model was 0.932, which is higher than that of the clinical-alone (0.897) or RNA-alone prognostic model (0.836). This clinical-RNA prognostic model can better classify the prognosis risk of EC patients. In the training group (396 patients), the overall survival of EC patients was lower in the high-risk group than in the low-risk group [HR = 32.263, (95% CI, 7.707–135.058), *P* = 8e-14]. The same comparison result was also observed for the validation group. A novel EC prognosis model integrating clinical variables and RNAs was established, which can better predict the prognosis and help to improve the clinical management of EC patients.

## Introduction

Endometrial carcinoma (EC) is a common malignant tumor of the female reproductive system, and its incidence is increasing (Chen W. et al., 2016; Siegel et al., 2020). Metastasis or recurrence often occurs in EC patients after surgery, and the median survival time of patients with recurrence or metastasis is generally <12 months (Obel et al., 2006). Chemotherapy and radiation therapy fail to kill tumor cells with high specificity. The 5-year overall survival rate of EC patients without metastasis is between 74 and 91% (Morice et al., 2016), while the rate is reduced to 68 or 17% for EC patients with local or distant metastases, respectively (Colombo et al., 2016). Therefore, it is urgent to study the factors and mechanisms that affect the prognosis of EC patients and improve the clinical management.

At present, the prognosis prediction of EC patients is mainly based on the age at diagnosis, FIGO stage, pathological classification, treatment method, and other clinical variables. Due to the strong individual differences in the stages of occurrence, development, and metastasis of EC, it is difficult to accurately predict the prognosis of EC patients through clinical variables only (Frederick and Straughn, 2009). Studies have shown that specific genes or molecular changes influence the prognosis of EC patients (Bell and Ellenson, 2019). Molecules such as *ER, PR, p53, HER-2/neu*, and *Ki-67* have been used to predict EC recurrence or prognosis; nevertheless, the results are still controversial (Fanning et al., 2002; Jeon et al., 2006).

In recent years, a class of non-coding RNA (ncRNA), including microRNA (miRNA) and long non-coding RNA (lncRNA), which cannot encode proteins, has been found to play an important role in life regulation (Djebali et al., 2012). More and more studies show that abnormal expression of ncRNA is closely related to the prognosis of EC patients. For example, *miRNA-200c, miR-944, HOTAIR, H19*, and *SRA* are related to prognosis of EC patients or the malignant degree of EC tumors (Smolle et al., 2015; He et al., 2017; Wilczynski et al., 2018). The expression levels of *miR-142-3p, miR-142-5p*, and *miR-15a-5p* are higher in EC patients with progression-free survival (PFS)>21 months than in EC patients with PFS <21 months, suggesting that *miR-142* and *miR-15a* may be useful for EC prognosis prediction (Jayaraman et al., 2017). *Hsa-mir-15a.MIMAT0000068, hsa-mir-142.MIMAT0000433, hsa-mir-142.MIMAT0000434, hsa-mir-3170.MIMAT0015045, hsa-mir-1976.MIMAT0009451*, and *hsa-mir-146a.MIMAT0000449* are significantly related to EC overall survival (OS), and the six-microRNA signature is an independent prognostic factor of EC (Wang Y. et al., 2019). However, so far, there has been no report on EC prognostic model integrating mRNAs, miRNAs, lncRNAs, and clinical variables.

This study intends to analyze the clinical and genome-wide mRNA, miRNA, and lncRNA expression data of EC patients in The Cancer Genome Atlas (TCGA) database and screen RNAs and clinical variables that are related to EC prognosis, with the expectation of discovering new EC prognostic molecular markers and establishing the integrated clinical-mRNA–miRNA–lncRNA prognostic model, thus providing a theoretical basis for EC prognostic risk assessment and individualized treatment.

## Materials and Methods

### Data Acquisition and Selection

We searched the TCGA database and other open databases, including Gene Expression Omnibus (GEO), International Cancer Genome Consortium (ICGC), ArrayExpress, Oncomine, etc. Only the TCGA database has an EC-related dataset with both gene profiles and clinical survival information. Clinical data, RNAseq-HTSeq FPKM data (including mRNA and lncRNA profiles), and miRNAseq data of EC patients were downloaded from the TCGA database (https://gdc-portal.nci.nih.gov/) in June 2019, and the dataset obtained contains 548 EC patients. Then, 52 of 548 EC patients were excluded. The reasons for exclusion were as follows: (1) 14 EC patients had no clinical data or mRNA, miRNA, and lncRNA gene expression profiles, and (2) 38 EC patients survived <30 days after the first pathological diagnosis. Eventually, 496 EC patients were included in this study, and the data missing rates for each clinical variable and the expression of each gene were <10%. The missing data of the 496 EC patients included in this study were filled by predictive mean matching. Differential expression analysis was performed based on log2 transformation of RNA expression data. From among all the patients involved in this study, 100 EC patients were randomly selected as the validation group, while the remaining 396 EC patients were used as the training group for the construction of the EC prognostic model. Furthermore, 33 out of the 496 EC patients had mRNA, miRNA, and lncRNA gene expression profiles of paracancerous tissues available, which were used as the control group for differential expression analysis.

### Construction of the Prognosis Model

The univariate Cox regression (the 2-sided log-rank test) was applied to analyze 12 clinical variables, including age at initial pathologic diagnosis, height, weight, histologic grade, clinical stage, histologic type, initial pathological diagnosis method, time since last menstruation, neoplasm status, race, surgical approach, and tissue indicator (prospective or retrospective). Clinical variables resulting in a univariate Cox regression *P* <0.05 were initially screened for inclusion multivariate Cox regression analysis (α_in_ = 0.10, α_out_ = 0.15). Then, the clinical variables selected by the multivariate Cox regression model were identified, and the prognosis clinical model (model 1) of EC patients based on the identified clinical variables was established. Clinical variables that had a hazard ratio (HR) for death>1 were considered to be risk-increasing clinical variables, and those with HR <1 were defined as protective clinical variables.

The RNA genes related to EC prognosis were screened by the following three steps: (1) A fold change (FC) and false discovery rate (FDR) were applied to identify RNAs with differential expression between the EC patient group (396 patients) and the control group (33 controls). mRNAs, miRNAs, and lncRNAs with a FC>2 or < -2 and FDR <0.05 were screened as differentially expressed RNAs. (2) Univariate Cox regression analysis was used to explore the relationship between the differentially expressed RNAs and the prognosis of EC patients, and the differentially expressed RNA with a univariate Cox regression *P* <0.05 is considered to be a prognosis-related RNA in EC patients. (3) The three types of RNAs (i.e., mRNA, miRNA, and lncRNA) with *P* <0.05 identified in the univariate Cox regression analysis were further subjected to elastic net-penalized Cox proportional hazards regression analysis with 10,000 iterations and 10 cross-validation (Zou and Hastie, 2005; Pak et al., 2020). Lastly, the mRNAs, miRNAs, and lncRNAs with a non-zero elastic net-penalized Cox proportional hazards regression coefficient were the final selected RNAs considered to be related to the OS of EC. Then, the ridge regression Cox model was used to fit the selected RNAs (mRNAs, miRNAs, and lncRNAs) to construct the prognosis models of mRNA (model 2), miRNA (model 3), and lncRNA (model 4), respectively. The integrated RNA molecular prognostic model (model 5) was then constructed by fitting the selected mRNAs, miRNAs, and lncRNAs with the ridge regression Cox model. Eventually, the integrated clinical-RNA prognostic model was established by fitting the screened prognosis-related clinical variables and RNAs with the ridge regression Cox model (model 6). RNAs that had a HR for death>1 were considered to be risk-increasing RNAs, and those with HR <1 were defined as protective RNAs.

### Evaluation of the Prognostic Model

The prognostic index (PI) is a weighted linear combination of various factors in the prognostic model. In the prognostic model, the PI value reflects the prognosis of the patient. PI is positively proportional to the risk function. A greater PI value indicates worse prognosis, and conversely, a smaller PI values means better prognosis. Standardization was carried out for PI to obtain a weighted prognostic index (WPI). The formulas used for calculating PI and WPI of each patient are as follows:

(1) $$\begin{array}{r} PI=\underset{i}{\sum }\left(\beta_{i}\times V_{i}\right) \end{array}$$

(2) $$\begin{array}{r} WPI=\frac{PI-mean\left(PI\right)}{SD\left(PI\right)}, \end{array}$$

Where β_i_ is the regression coefficient of the i-th factor in the model, *V*_i_ is the value of the i-th factor of EC patients, and mean (PI) and SD (PI) are the mean and standard deviation (SD) of the PI vector in EC patients, respectively. Applying WPI = 0 as the cutoff point, the patients were classified into two groups in terms of the predicted prognosis. Specifically, patients with WPI ≤ 0 were in the low-risk group, whereas those with WPI > 0 were in the high-risk group. The Kaplan–Meier curves of patients in the high-risk group and low-risk group were drawn and subjected to the log-rank test. *P* ≤ 0.05 indicates statistically significant difference in the OS between the two groups. The areas under the time-dependent ROC curves (AUC-ROC) of the six prognostic models were calculated. The model with the greatest AUC value was selected as the optimal prognostic model. An AUC value between 0.7 and 0.9 is generally believed to indicate medium predictive ability, while an AUC value greater than 0.9 indicates relatively ideal predictive ability. The larger is the AUC value, the stronger is the predictive ability of the model.

### GO and KEGG Enrichment Analysis

The online tool DAVID (The Database for Annotation, Visualization and Integrated Discovery, version 6.8, http://david.abcc.ncifcrf.gov) was used to perform the GO and KEGG enrichment analysis for mRNAs, miRNA-targeted mRNAs (mRNAs with miRDB database-predicted scores higher than 90), and lncRNA-related mRNAs (Spearman's correlation coefficient *r*_s_>0.50 and *P* <0.05) in the EC prognostic molecular model. Fisher's exact test was employed to select terms with *P* <0.05 as significant GO and KEGG pathway terms. GO analysis annotates and classifies genes through biological process (BP), molecular function (MF), and cell composition (CC).

### Statistical Analysis

Data analyses in this study were conducted by R, version 3.6.1. The missing data were filled by the “mice” R package (version 3.11.0), and differential expression analysis was performed with the “limma” R package (version 3.26.9). The “survival” R package (version 3.2-3) was applied for univariate Cox regression, multivariate Cox regression, and plotting Kaplan–Meier curves. The elastic net-penalized Cox proportional hazards regression model and the ridge regression Cox model were analyzed using the “glmnet” R package (version 3.0-2). The “timeROC” R package (version 0.4) was used to plot the time-dependent ROC curves and calculate the AUC values, and the “ggplot2” R package (version 3.3.1) was used to generate figures of GO and KEGG analysis.

## Results

### Workflow

**Figure 1:**
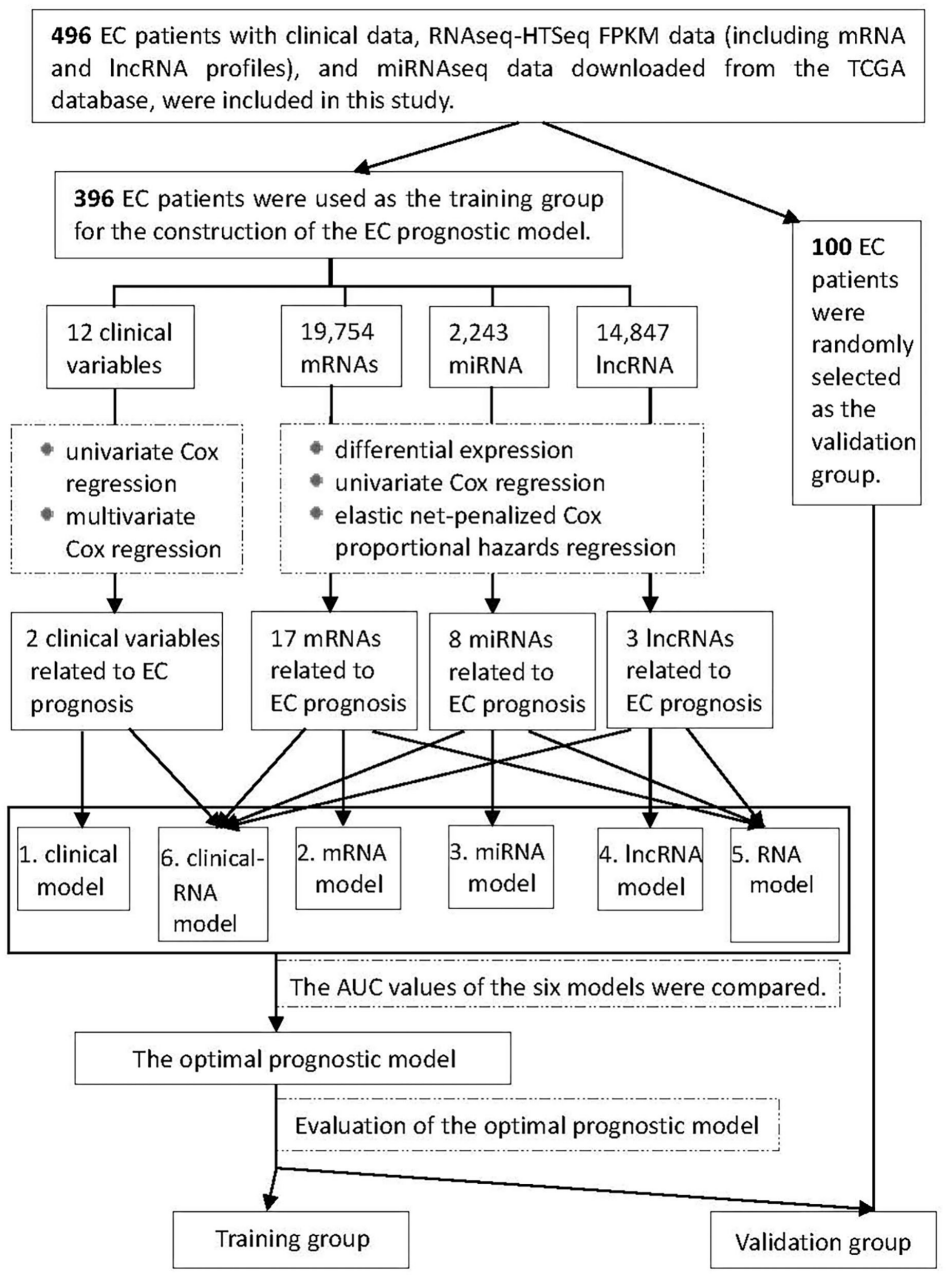
Flowchart of construction and evaluation of the EC prognostic model. AUC, the area under the receiver operating characteristic curve.

Figure 1 shows the process of our Study. RNA expression data and corresponding clinical variable data from TCGA for EC were analyzed. Cox proportional hazards regression was used to analyze clinical variables related to EC prognosis. RNAs related to EC prognosis were screened by differential expression analysis, univariate Cox proportional hazards regression, and elastic net-penalized Cox proportional hazards regression. The EC prognostic model was constructed by using EC prognostic-related RNAs/clinical variables, and the performance of the prognostic model was evaluated.

### Clinical Characteristics and Prognosis Model of EC Patients

Among the 396 EC patients in the training group, 33 patients died by the follow-up deadline and 363 patients survived. The minimum and maximum ages of patients at initial pathological diagnosis were 33 years and 89 years, respectively, with the average age being 64.22 years (*SD* = 10.86 years). Univariate Cox regression analysis indicated that histological grade, clinical stage, and neoplasm status were statistically significant (*P* <0.05) among the 12 clinical variables. Further multivariate Cox regression analysis was performed for the three factors, and the results suggested that histological grade and neoplasm status are independent prognostic clinical variables of EC, and both of them are EC risk factors (HR > 1). Then, a clinical prognostic model of EC was established based on histological grade and neoplasm status (Table 1).

**Table 1:** Survival analysis results of demographic and clinical variables for EC patients in the prognostic model training group (396 patients).

**Variables**	***n* (%)^a^**	**Univariate Cox**		**Multivariate Cox^c^**	
		**HR^b^ (95% CI)**	***P-*value**	**HR^b^ (95% CI)**	***P-*value**
Histological grade		3.27 (1.48–7.23)	0.003	1.89 (0.87–4.10)	0.107
G1	70 (17.68)				
G2	88 (22.22)				
G3	238 (60.10)				
Neoplasm status		22.45 (9.25–54.47)	<0.001	18.24 (7.38–45.08)	<0.001
Yes	319 (84.62)				
No	58 (15.38)				
Data missing	19				
Clinical stage		2.20 (1.61–2.98)	<0.001		
I	241 (60.86)				
II	42 (10.60)				
III	95 (23.99)				
IV	18 (4.55)				
Age at initial pathological diagnosis (years)	1.69 (0.78–3.63)	0.182		
≤ 60	143 (36.11)				
>60	253 (63.89)				
Height (cm)		1.13 (0.84–1.53)	0.418		
≤ 155	77 (20.70)				
≤ 160	102 (27.42)				
≤ 165	95 (25.54)				
>165	98 (26.34)				
Data missing	24				
Weight (kg)		1.01 (0.73–1.39)	0.969		
≤ 50	118 (31.13)				
≤ 60	101 (26.65)				
≤ 70	85 (22.43)				
>70	75 (19.79)				
Data missing	17				
Histological type^d^					
Serous	88 (22.22)	–	0.111		
Endometrioid	294 (74.24)	1.19 (0.15–9.22)	0.867		
Mixed serous and endometrioid	14 (3.54)	0.56 (0.07–4.17)	0.568		
Initial pathological diagnosis method	1.82 (0.91–3.65)	0.093		
Biopsy	248 (63.42)				
Other	143 (36.58)				
Data missing	5				
Time since last menstruation (months)	0.76 (0.44–1.31)	0.323		
≤ 6	25 (6.87)				
≤ 12	9 (2.47)				
>12	330 (90.66)				
Data missing	32				
Race^d^					
Black	87 (23.32)	–	0.649		
White	260 (69.71)	0.39 (0.08–2.00)	0.257		
Asian	17 (4.56)	0.45 (0.11–1.90)	0.277		
Other	9 (2.41)	0.31 (0.04–2.25)	0.249		
Data missing	23				
Surgical approach		1.26 (0.61–2.63)	0.532		
Open surgery	222 (59.04)				
Minimally invasive	154 (40.96)				
Data missing	20				
Tissue collection indicator		0.80 (0.18–3.53)	0.769		
Prospective	79 (19.95)				
Retrospective	317 (80.05)				

a *Before missing data is filled*.

b *Protective RNA had a HR <1 and risky RNA had a HR > 1 in EC patients*.

c *The multivariate Cox regression analysis (α_in_ = 0.10, α_out_ = 0.15) was carried out for clinical variables with P <0.05 in the univariate Cox regression analysis*.

d *Dummy variables were applied*.

*EC, endometrial carcinoma; CI, confidence interval; HR, hazard ratio*.

### Differentially Expressed and OS-Related RNAs of EC

**Figure 2:**
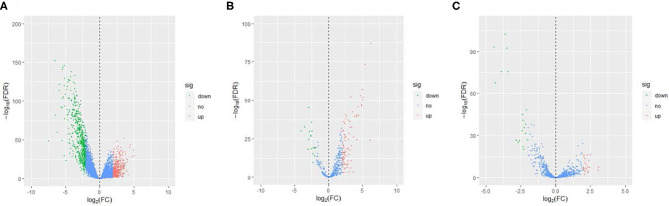
Volcano diagram of the differential expression RNAs in ECs and controls. **(A)** Volcano diagram of mRNA, **(B)** volcano diagram of miRNA, and **(C)** volcano diagram of lncRNA.

**Figure 3:**
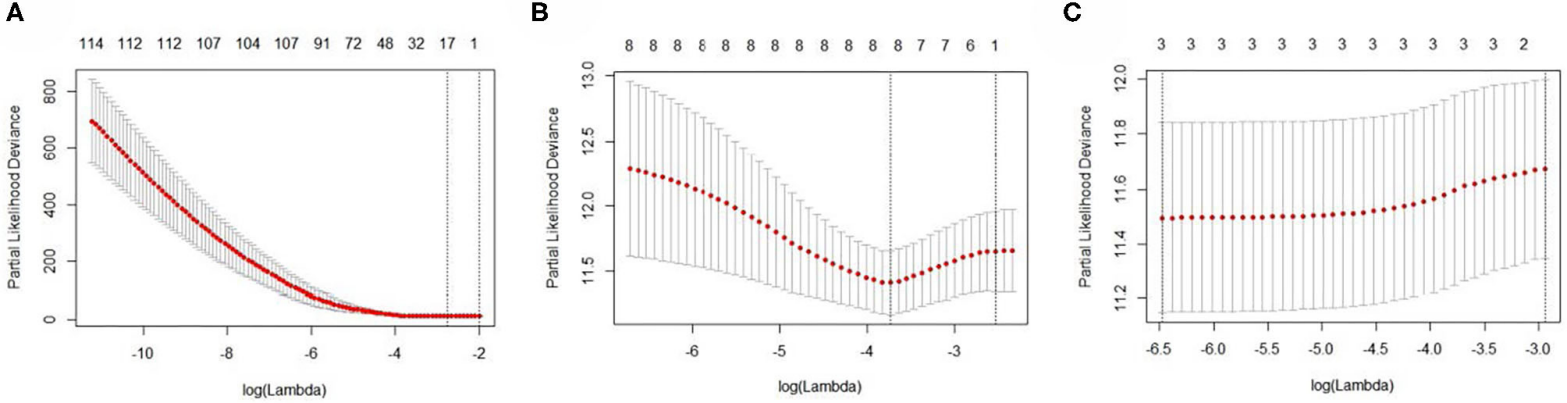
The cross-validation curves of the elastic net-penalized Cox proportional hazard regression. **(A)** Cross-validation curve for mRNA, **(B)** cross-validation curve for miRNA, and **(C)** cross-validation curve for lncRNA.

The EC RNA expression data acquired from TCGA database were preprocessed, and a total of 36,844 RNAs (19,754 mRNA, 2,243 miRNA, and 14,847 lncRNA) were included in this study. 1060 differential expression RNAs were screened by differential expression analysis, including 920 mRNAs (353 upregulation and 567 downregulation, Figure 2A), 100 miRNAs (83 upregulation and 17 downregulation, Figure 2B), and 40 lncRNAs (21 upregulation and 19 downregulation, Figure 2C). Univariate Cox regression was performed on these 1,060 differentially expressed RNAs individually, and there were 126 RNAs with *P* <0.05 (115 mRNAs, 8 miRNAs, and 3 lncRNAs). Subsequently, the whole 126 RNAs were subjected to elastic net-penalized Cox proportional hazard regression analysis, and 17 RNAs related to EC prognosis were selected, including 15 mRNAs (*ANGPTL1, ALDH1A1, FIBIN, GFPT2, HIST1H3H, HOXD8, IGFBP5, MAL, MMP1, PRKAR2B, PROM2, SCARA3, SNAP25, TFPI*, and *TSPYL5*), and 2 miRNAs (*has-miR-215-5p* and *has-miR-592*). There is no lncRNA in the 17 RNAs. We do not think it can reflect the whole picture of RNAs associated with EC prognosis. Recent studies have shown that although lncRNA does not encode proteins, lncRNA participates in gene expression regulation at various levels, such as transcriptional regulation and posttranscriptional regulation. The abnormal expression of lncRNA is usually associated with the occurrence, recurrence, and metastasis of tumors (Kopp and Mendell, 2018). Therefore, we used elastic net-penalized Cox proportional hazard regression to screen the three types of RNAs (mRNA, miRNA, and lncRNA), respectively. Eventually, 28 EC OS-related RNAs (17 mRNAs, 8 miRNAs, and 3 lncRNAs) were identified (Figure 3, Table 2). These 28 EC OS-related RNAs contain all 17 RNAs screened by elastic net-penalized Cox proportional hazard regression using whole genes.

**Table 2:** The 28 identified EC prognosis-related RNAs.

**Number**	**Gene symbol**	**HR^a^**	**95% CI**	***P*-value^b^**	**Regulation^c^**	**Coefficient^d^**
**mRNA**						
1	*ALDH1A1*	1.001	1.001–1.002	<0.001	Down	0.0002
2	*ANGPTL1*	1.001	1.001–1.002	<0.001	Down	0.0018
3	*COL4A6*	1.113	1.061–1.169	<0.001	Down	0.0324
4	*FIBIN*	1.028	1.016–1.040	<0.001	Down	0.0024
5	*GFPT2*	1.026	1.014–1.038	<0.001	Down	0.0042
6	*HIST1H3H*	1.011	1.005–1.016	<0.001	Up	0.0059
7	*HOXD8*	1.019	1.009–1.028	<0.001	Down	0.0022
8	*IGFBP5*	1.001	1.0005–1.0014	<0.001	Down	0.0001
9	*MAL*	1.001	1.001–1.002	<0.001	Up	0.0005
10	*MMP1*	1.006	1.003–1.009	<0.001	Up	0.0005
11	*PRKAR2B*	1.036	1.016–1.055	<0.001	Down	0.0034
12	*PROM2*	1.008	1.004–1.012	<0.001	Up	0.0031
13	*RAB26*	1.020	1.008–1.032	0.001	Up	0.0001
14	*SCARA3*	1.003	1.002–1.005	<0.001	Down	0.0011
15	*SNAP25*	1.125	1.075–1.177	<0.001	Down	0.0567
16	*TFPI*	1.057	1.030–1.084	<0.001	Down	0.0114
17	*TSPYL5*	1.015	1.008–1.022	<0.001	Down	0.0078
**miRNA**						
18	*hsa-miR-141-3p*	1.034	1.001–1.068	0.041	Up	−0.0002
19	*hsa-miR-191-5p*	0.999	0.999–1.000	0.048	Up	−0.00002
20	*hsa-miR-192-5p*	1.000	1.00001–1.00007	0.011	Up	0.00002
21	*hsa-miR-215-5p*	1.003	1.001–1.005	<0.001	Up	0.00205
22	*hsa-miR-3170*	0.875	0.779–0.983	0.024	Up	−0.0465
23	*hsa-miR-3613-5p*	0.963	0.931–0.997	0.034	Up	−0.0143
24	*hsa-miR-592*	1.006	1.003–1.009	0.001	Up	0.00483
25	*hsa-miR-7-5p*	1.034	1.001–1.068	0.041	Up	0.03034
**lncRNA**						
26	*DNM3OS*	1.057	1.004–1.112	0.036	Down	0.05966
27	*FAM83H-AS1*	1.014	1.001–1.027	0.042	Up	0.01465
28	*RP11-295G20.2.1*	1.010	1.000–1.020	0.049	Up	0.00946

a *Protective RNA had a HR <1 and risky RNA had a HR > 1 in EC patients*.

b *Univariate Cox regression P value <0.05 was considered statistically significant*.

c *Type of regulation (upregulated or downregulated) in ECs vs controls*.

d *Elastic net-regulated Cox regression coefficient*.

*EC, endometrial carcinoma; HR, hazard ratio; CI, confidence interval*.

### RNA Molecular Prognostic Models of EC

The EC OS-related 17 mRNAs, 8 miRNAs, and 3 lncRNAs were fitted with the ridge regression Cox model respectively to obtain the corresponding mRNA prognostic model, miRNA prognostic model, and lncRNA prognostic model. The 28 EC OS-related RNAs were fitted with the ridge regression Cox model, and an integrated mRNA–miRNA–lncRNA molecular prognostic model was established (Table 3).

**Table 3:** Ridge regression Cox prognostic model.

**Number**	**Variable**	**Coefficient^a^**	**Coefficient^b^**	**Coefficient^c^**	**Coefficient^d^**	**Coefficient^e^**
**mRNA**					
1	*ALDH1A1*	0.000460	-	-	0.0003	0.0003
2	*ANGPTL1*	0.005925	-	-	0.0056	0.0033
3	*COL4A6*	0.033186	-	-	0.0322	0.0343
4	*FIBIN*	0.004171	-	-	0.0053	0.0037
5	*GFPT2*	0.005855	-	-	0.0058	0.0033
6	*HIST1H3H*	0.006833	-	-	0.0068	0.0071
7	*HOXD8*	0.003590	-	-	0.0039	0.0034
8	*IGFBP5*	0.000262	-	-	0.0003	0.0003
9	*MAL*	0.000456	-	-	0.0004	0.0005
10	*MMP1*	0.001393	-	-	0.0011	0.0004
11	*PRKAR2B*	0.009734	-	-	0.0097	0.0043
12	*PROM2*	0.002367	-	-	0.0020	0.0018
13	*RAB26*	0.005232	-	-	0.0051	0.004
14	*SCARA3*	0.001498	-	-	0.0014	0.0019
15	*SNAP25*	0.044875	-	-	0.0377	0.0333
16	*TFPI*	0.015739	-	-	0.0141	0.0173
17	*TSPYL5*	0.008143	-	-	0.0079	0.0066
**miRNA**					
18	*hsa-miR-141-3p*	-	−0.000121699	-	−0.00006	−0.00007
19	*hsa-miR-191-5p*	-	−0.000123281	-	−0.00004	−0.00003
20	*hsa-miR-192-5p*	-	2.08217E-05	-	0.00001	0.00001
21	*hsa-miR-215-5p*	-	0.001517209	-	0.0009	0.0009
22	*hsa-miR-3170*	-	−0.02585058	-	−0.019	−0.0256
23	*hsa-miR-3613-5p*	-	−0.00941356	-	−0.0058	−0.0063
24	*hsa-miR-592*	-	0.00339596	-	0.0033	0.005
25	*hsa-miR-7-5p*	-	0.01726421	-	0.0144	0.0159
**lncRNA**					
26	*DNM3OS*	-	-	0.054349555	0.0044	0.0074
27	*FAM83H-AS1*	-	-	0.012946912	0.0023	0.0021
28	*RP11-295G20.2.1*	-	-	0.008863742	0.0034	0.0042
**Clinical**					
29	Histologic grade	-	-	-	-	0.1432
30	Neoplasm status	-	-	-	-	1.1664

a *Coefficient of the mRNA ridge regression Cox model (model 2)*.

b *Coefficient of the miRNA ridge regression Cox model (model 3)*.

c *Coefficient of the lncRNA ridge regression Cox model (model 4)*.

d *Coefficient of the mRNA–miRNA–lncRNA ridge regression Cox model (model 5)*.

e *Coefficient of the clinical-RNA ridge regression Cox model (model 6)*.

### Integrated Clinical-RNA Prognostic Model of EC

**Figure 4:**
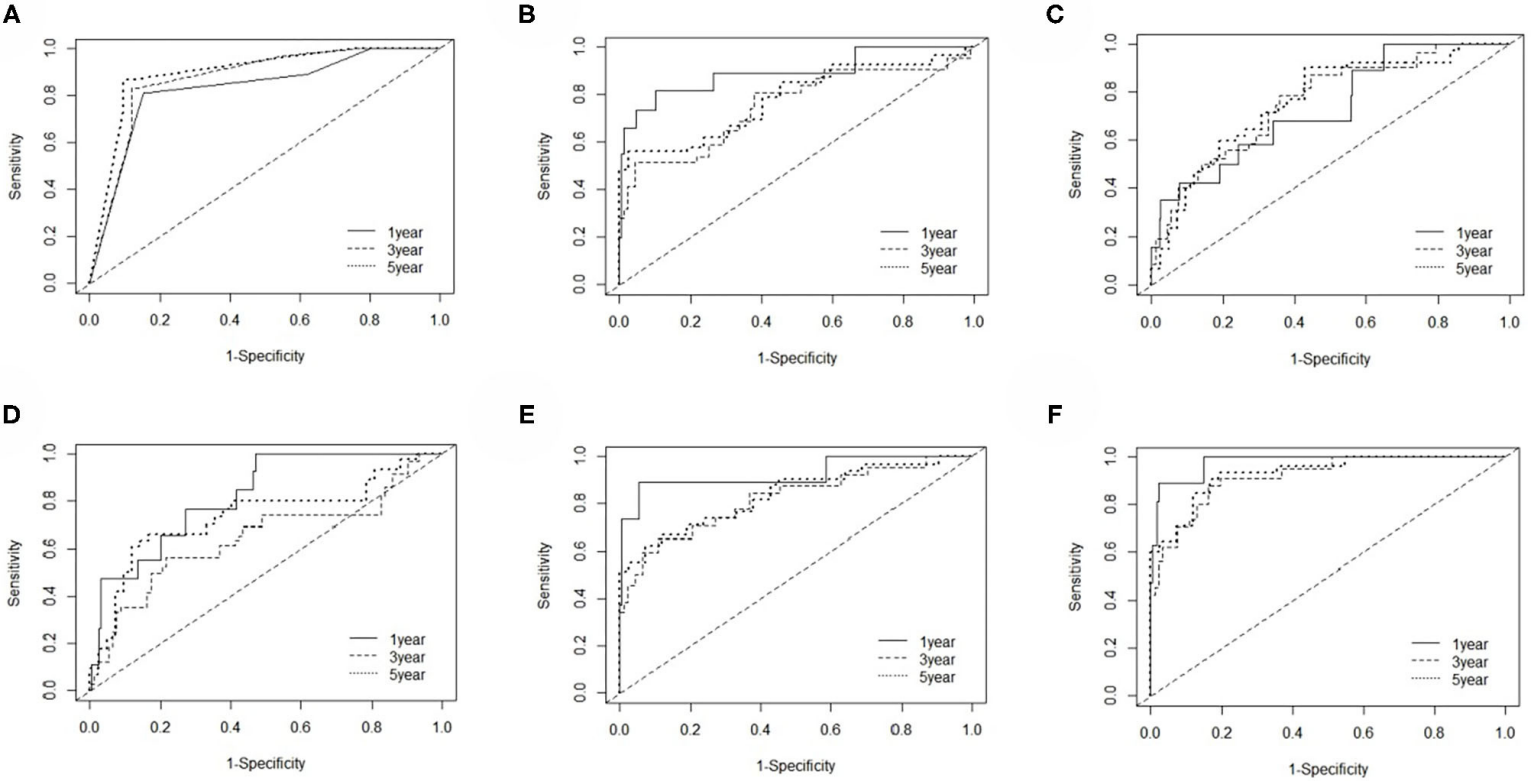
The time-dependent receiver operating characteristic curves. **(A**–**F)** The 1-, 3-, and 5-year ROC curves of the clinical model, mRNA model, miRNA model, lncRNA model, RNA model, and clinical-RNA model.

The EC prognosis-related 28 RNAs and 2 clinical variables were fitted with the ridge regression Cox model, and an integrated clinical-RNA prognostic model was established (Table 3). As listed in Table 4, the AUC (95% CI) values of the lncRNA model based on the selected three lncRNAs at 1, 3, and 5 years were 0.823 (0.719–0.928), 0.646 (0.505–0.788), and 0.737 (0.608–0.870), respectively. These results suggest that the three lncRNAs have a certain predictive effect on the prognosis of EC. The AUC-ROC showed that except for the lncRNA molecular model (model 4) with a 3-year prognosis AUC of 0.646 (<0.7), the minimum AUC value of other models was 0.733. The AUC of the integrated RNA molecular prognostic model (model 5) was ≥0.821, which is greater than the AUC of mRNA, miRNA, and lncRNA models, suggesting that the integrated RNA molecular prognostic model is superior to the mRNA or miRNA or lncRNA model in terms of predictive ability. As for the clinical prognostic model, the AUC value was ≥0.830, implying that the two clinical variables (i.e., histological grade and neoplasm status) screened in this study can predict the prognosis of EC. The 1-, 3-, and 5-year AUC values of the integrated clinical-RNA prognostic model (model 6) were ≥0.919, being greater than the AUC values of other models at the same time point. This indicates that the integrated clinical-RNA prognostic model has the best predictive ability among the six models (Figure 4).

**Table 4:** 1-, 3-, and 5-year AUC of the EC prognostic model.

**Model**	**1-year**		**3-year**		**5-year**	
	**AUC**	**95% CI**	**AUC**	**95% CI**	**AUC**	**95% CI**
1. Clinical model	0.830	0.695–0.964	0.872	0.808–0.936	0.897	0.828–0.966
2. mRNA model	0.894	0.764–1.025	0.756	0.634–0.879	0.783	0.664–0.903
3. miRNA model	0.733	0.579–0.888	0.761	0.653–0.869	0.763	0.646–0.879
4. lncRNA model	0.823	0.719–0.928	0.646	0.505–0.788	0.737	0.608–0.870
5. RNA model	0.927	0.813–1.042	0.821	0.717–0.925	0.836	0.733–0.938
6. Clinical-RNA model	0.979	0.949–1.008	0.919	0.860–0.978	0.932	0.875–0.989

*AUC, the area under the receiver operating characteristic curve; CI, confidence interval*.

**Figure 5:**
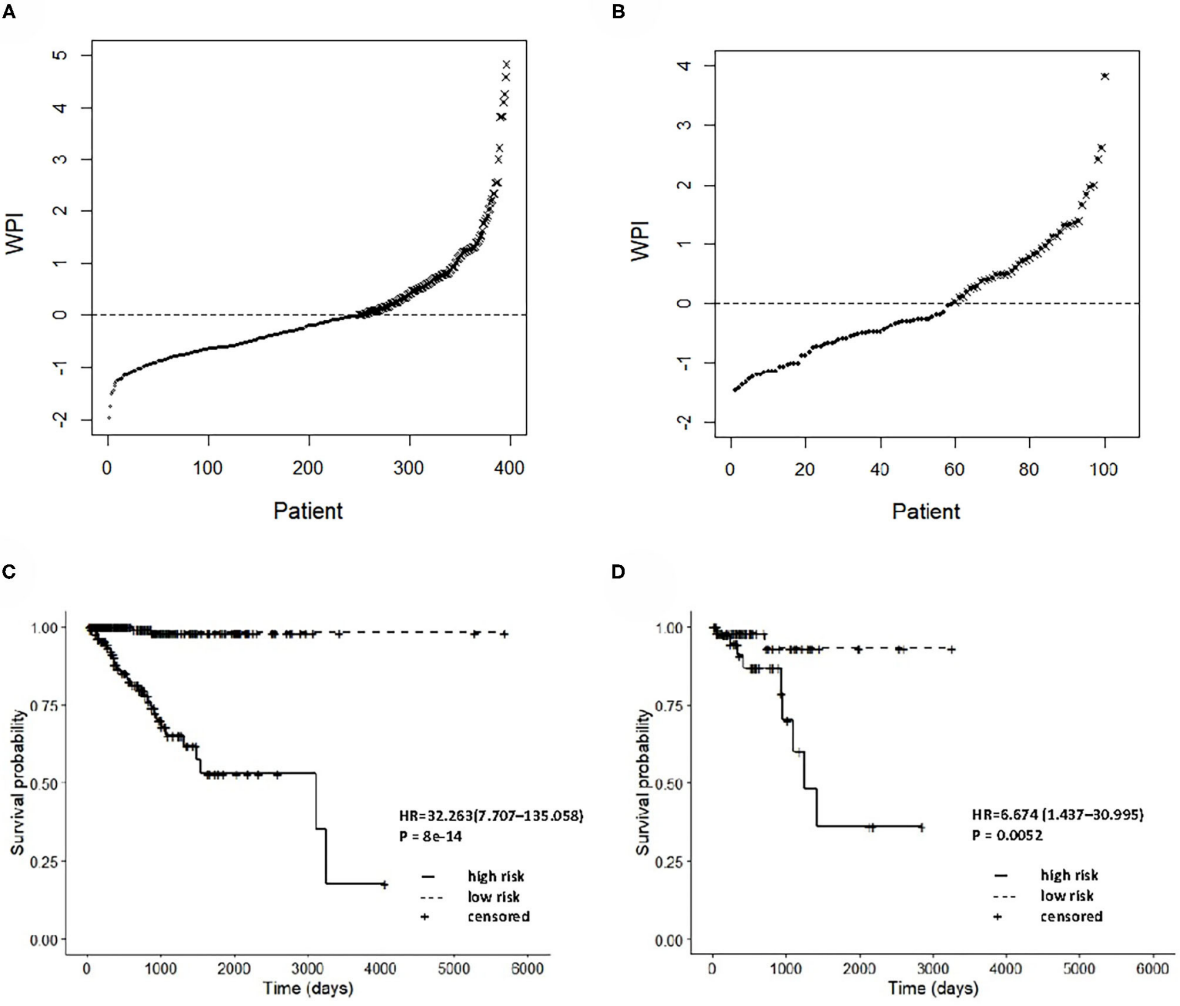
Prognostic performance assessment of integrated clinical-RNA prognostic model. WPI distributions and stratifications of EC patients in training set **(A)** and validating set **(B)**, respectively. Kaplan–Meier curves for stratifications in training set **(C)** and validating set **(D)**, respectively. WPI, weighed prognostic index; HR, hazard ratio.

The integrated clinical-RNA prognostic model was used to calculate the WPI value of each EC patient in the training group (396 patients). The WPI value of EC patients ranged from −1.957 to 4.831. Taking WPI = 0 as the cutoff point, the EC patients were divided into the high-risk group (147 patients) and low-risk group (249 patients) (Figure 5A). The difference between the two groups' Kaplan–Meier curves was statistically significant (*P* = 8e-14). The prognosis of EC patients was worse in the high-risk group than in the low-risk group [HR = 32.263, (95% CI, 7.707–135.058)], suggesting that the integrated clinical-RNA prognostic model enables accurate prediction of the prognosis of EC patients (Figure 5C). Furthermore, this model was used to calculate the WPI value of each EC patient in the validation group (100 patients) to predict the prognosis of EC patients. The WPI value of EC patients in the validation group ranged from −1.455 to 3.822. Taking WPI = 0 as the cutoff point, the EC patients in the validation group were divided into the high-risk group (41 patients) and low-risk group (59 patients) (Figure 5B). The difference between the two groups' Kaplan–Meier curves was statistically significant (*P* = 0.0052). The prognosis of EC patients in the high-risk group was worse than that of patients in the low-risk group [HR = 6.674, (95% CI, 1.437–30.995)], showing that the integrated clinical-RNA prognostic model also has satisfactory accuracy in predicting the prognosis of EC patients in the validation group (Figure 5D).

### Functional Analysis of EC Prognosis-Related Genes

**Figure 6:**
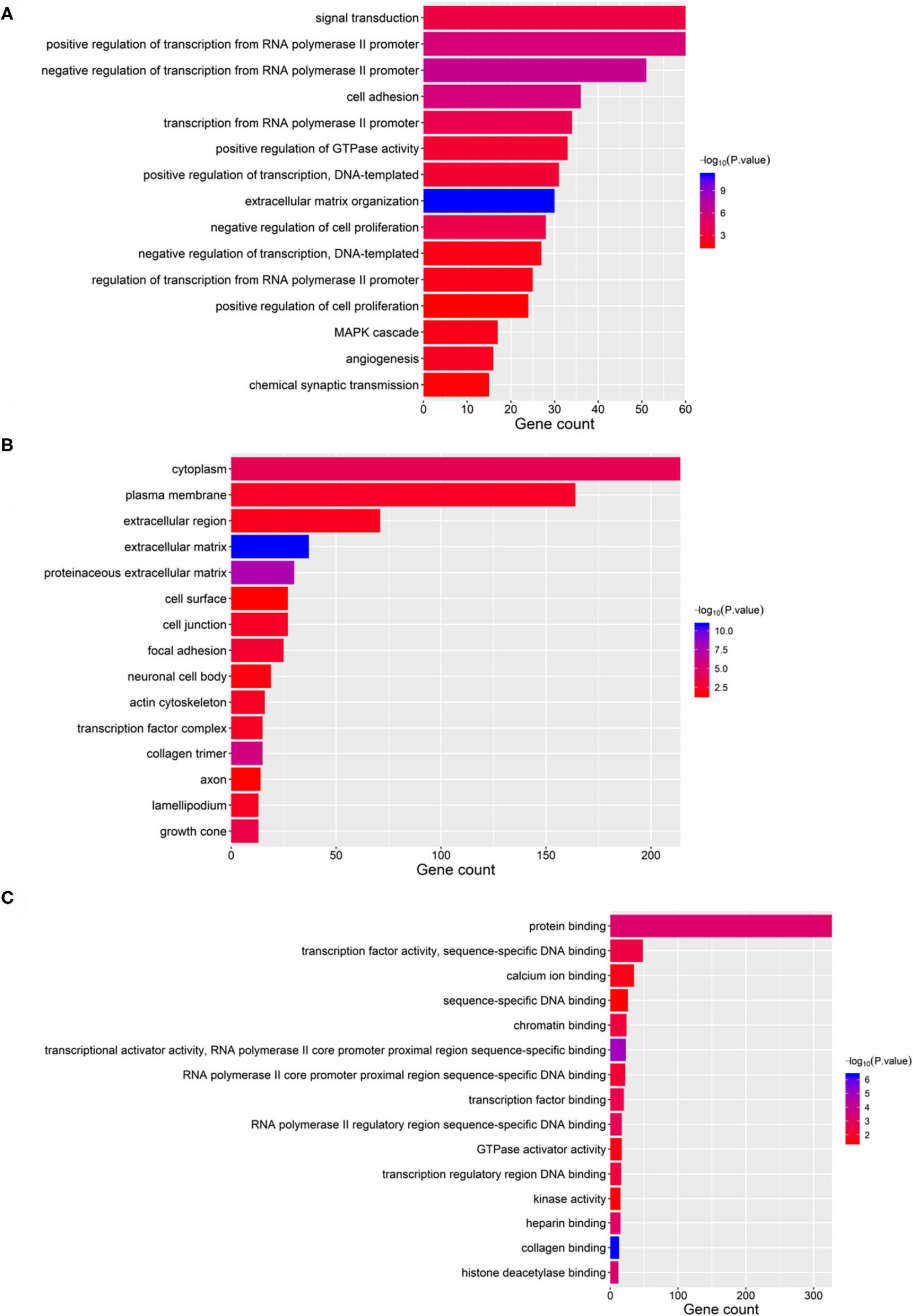
GO analysis of the 28 validated genes. **(A)** Biological process (BP), **(B)** cellular component (CC), and **(C)** molecular function (MF).

**Figure 7:**
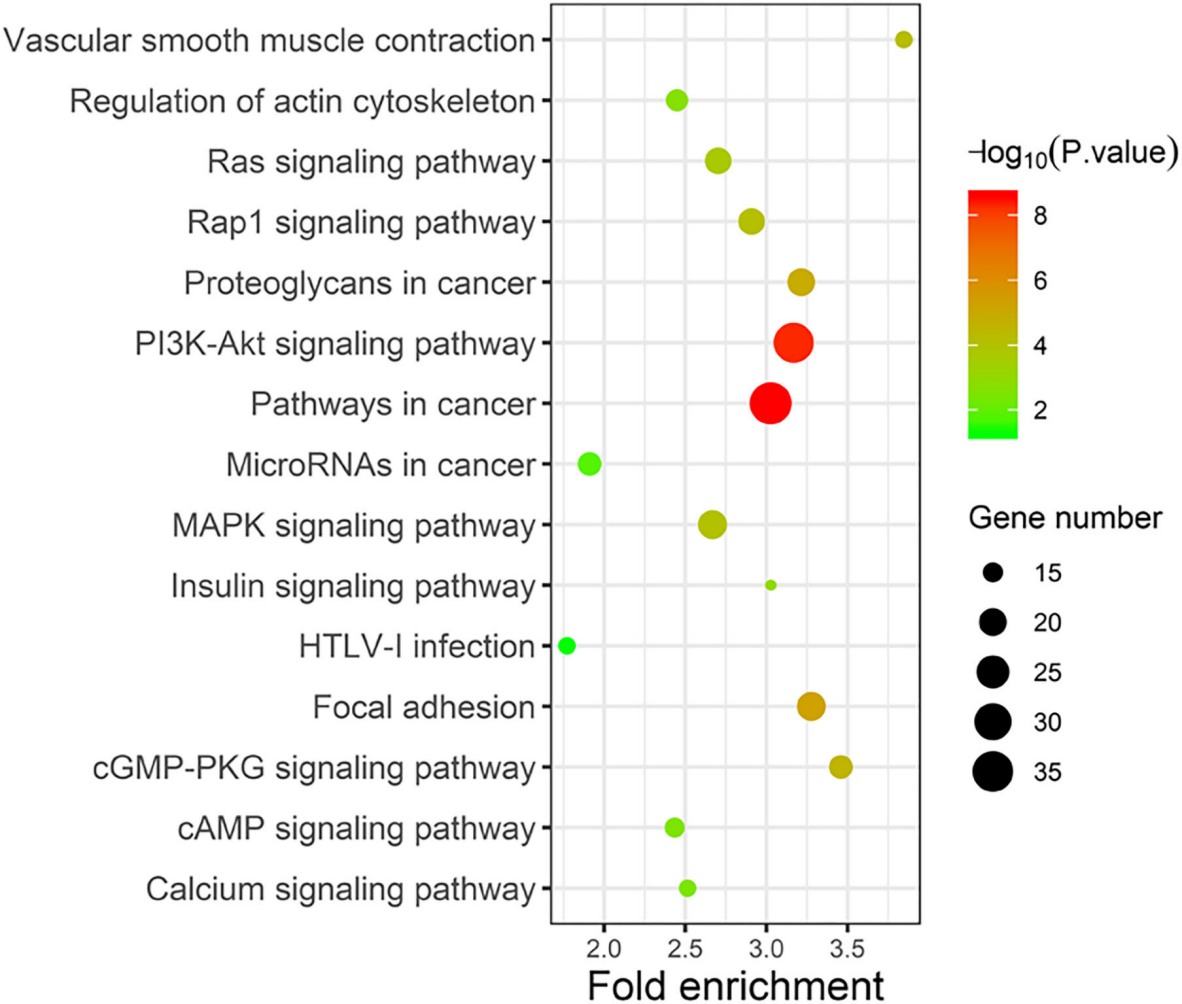
KEGG analysis of the 28 validated genes.

Taking the union of the 17 mRNAs, the 371 target mRNAs of the 8 miRNAs, and the 300 mRNAs related to the 3 lncRNAs in the RNA prognostic model, a gene set with 652 mRNAs was obtained and subjected to GO and KEGG analyses. The GO analysis results show that in biological processes, target genes are mainly enriched in “signaling,” “positive regulation of RNA polymerase II promoter transcription,” and “negative regulation of RNA polymerase II promoter transcription” (Figure 6A). In terms of cellular composition, the target genes are mainly located in the “cytoplasm” and “plasma membrane” (Figure 6B). As for the molecular function, “protein binding” is the most important mode (Figure 6C). According to the results of KEGG analysis, the top three pathways are pathways in cancer, the PI3K-Akt signaling pathway, and the focal adhesion pathway (Figure 7).

## Discussion

At present, researchers have been searching for EC prognosis-related biomarkers and establishing EC prognostic prediction model with higher accuracy to provide better clues for formulating reasonable individualized treatment plans, thereby improving patients' prognostic quality of life. After acquiring the clinical data of EC patients from the TCGA database and the full mRNA, miRNA, and lncRNA genome expression profiles, 30 factors related to EC prognosis, including two clinical variables and 28 RNAs, were identified in this study. Based on the 30 EC prognosis-related factors, 6 EC prognosis models were established: clinical model, mRNA model, miRNA model, lncRNA model, integrated RNA model, and integrated clinical-RNA model. The clinical-RNA model displayed the highest AUC (≥ 0.919), indicating the strongest predictive ability among the six prognostic models.

Previous studies have shown that the clinical variables related to the prognosis of EC include pathological grade, pathological stage, FIGO stage, age at initial pathological diagnosis, degree of muscular invasion, vascular tumor thrombus, and lymph node metastasis (Braun et al., 2016; Morice et al., 2016). Our study suggests that histological grade and neoplasm status are independent prognostic factors for EC overall survival.

There were 17 prognosis-related mRNAs with HR>1, indicating that an increased expression level of these mRNAs will increase the risk of death in EC patients. Most of these genes are reportedly related to the occurrence, development, or prognosis of cancer. The expression of *ALDH1A1* is upregulated in endometrial carcinoma cells (Shiba et al., 2019), and *ALDH1A1* is a confirmed oncogene for lung cancer (Gao et al., 2015). As a member of angiopoietin-like protein genes, *ANGPTL1* acts as a tumor-suppressor gene in various tumors (Chen H. A. et al., 2016). The absence of *COL4A6* may cause familial hemorrhagic nephritis (Murata et al., 2016). *GFPT2* is highly expressed in lung cancer (Zhang et al., 2018). *HIST1H3H* is a histone gene, and its high expression is related to the OS, relapse-free survival (RFS), and distant metastasis-free survival (DMFS) of breast cancer patients (Xie et al., 2019). *HOXD8* belongs to a homeobox gene family and is closely related to cell proliferation, apoptosis, and cell cycle. Studies have found that *HOXD8* is a downstream target gene of *miR-5692a*. *MiR-5692a* plays the role of an oncogene in the occurrence and development of liver cancer by regulating the expression of *HOXD8* (Sun et al., 2019). *IGFBP5* is a tumor-suppressor gene for leukemia, osteosarcoma, breast cancer, and pancreatic cancer and participates in cell biological functions, such as cell metastasis and apoptosis (Baxter, 2014). Hypermethylation of *MAL* in cervical intraepithelial neoplasia accelerates cervical lesions (Meršaková et al., 2018). High expression of *MMP1* in cancer tissues leads to accelerated angiogenesis, thus promoting the proliferation and migration of cancer cells (Pahwa et al., 2014). It has been clarified that the *PRKAR2B* gene is overexpressed in castration-resistant prostate cancer (CRPC), which mainly promotes cell-cycle biological processes, accelerates CRPC cell proliferation and invasion, and inhibits CRPC cell apoptosis (Sha et al., 2017). The expression of *PROM2* (prominin 2) is upregulated in kidney cancer and melanoma (Rohan et al., 2006; Winnepenninckx et al., 2006). The function of *RAB26* is mainly related to membrane transport and cell autophagy. Some studies have found that *RAB26* can affect the metastasis and invasion of breast cancer (Schwartz et al., 2007). *SCARA3* inhibits the lethal effect of dexamethasone and bortezomib on myeloma cells (Brown et al., 2013). *SNAP25* is mainly involved in the occurrence and development of mental diseases (González-Giraldo and Forero, 2020). *TFPI* reduces tumor cell-induced coagulation activation and lung metastasis, and it has shown inhibitory effect on primary and metastatic tumors in mice (Hembrough et al., 2003; Amirkhosravi et al., 2007). Some studies suggest that the methylation of the tumor-suppressor gene *TSPYL5* will cause its expression silencing and, thereby, gastric cancer (Jung et al., 2008).

In this study, eight EC prognosis-related miRNAs were identified. The HR values of *miR-141-3p, miR-192-5p, miR-215-5p, miR-592*, and *miR-7-5p* were greater than 1, indicating that the five miRNAs are highly expressed in EC, acting as tumor genes and prognostic risk factors. *MiR-141-3p* acts as a tumor-suppressor gene in colorectal cancer and enhances the sensitivity of colorectal cancer cells to cetuximab by inhibiting *EGFR* (Xing et al., 2020). *MiR-192-5p* plays different roles in different cancers, e.g., it is highly expressed in gastric cancer and pancreatic ductal cancer, while the expression is low in lung cancer (Feng et al., 2011; Zhao et al., 2013; Chen et al., 2014). Overexpression of *miR-215-5p* in colorectal cancer leads to G2/M phase cell-cycle arrest and *p53*-dependent apoptosis induction, thus reducing the proliferation and migration of colorectal cancer cells (Vychytilova-Faltejskova et al., 2017). The biological function of *miR-592* varies according to the cancer type. Its overexpression in liver cancer inhibits the proliferation and metastasis of cancer cells, while the opposite effect is observed in prostate cancer (Wang et al., 2012; Lv et al., 2015). Studies have shown that *miR-7-5p* can inhibit tumor development by regulating the PI3K/Akt pathway and the expression of the target gene *KLF4* (Fang et al., 2012; Okuda et al., 2013). The other three miRNAs (i.e., *miR-191-5p, miR-3170*, and *miR-3613-5p*) have HR values lower than 1, indicating that these three genes are protective factors for EC prognosis, i.e., their high expression reduces the risk of death in EC patients. The overexpression of *miR-191-5p* in lung adenocarcinoma downregulates Wnt signaling via the target gene *SATB1*, thus blocking lung cancer cell migration and proliferation (Zhou et al., 2020). Studies have shown that the prognosis is better in EC patients with high expression of *miR-3170* than in those with low expression of *miR-3170* (Wang Y. et al., 2019). The expression level of *miR-3613-5p* in the serum of patients with endometriosis is significantly reduced (Cosar et al., 2019).

The HR values of the three identified EC prognosis-related lncRNAs (e.g., *DNM3OS, FAM83H-AS1*, and *RP11-295G20.2.1*) are all greater than 1, indicating that they are prognostic risk factors for EC. Studies have observed that the expression of *DNM3OS* is upregulated in gastric cancer tissues and cell lines. Knocking out *DNM3OS* hinders snail-mediated epithelial-to-mesenchymal transition, thereby inhibiting the proliferation, migration, and invasion of gastric cancer cells (Wang S. et al., 2019). *FAM83H-AS1* promotes radiation resistance and metastasis of ovarian cancer via targeted HuR protein (Dou et al., 2019). At present, the specific biological function of *RP11-295G20.2.1* is not clear, and its relationship with the occurrence, development, and prognosis of EC needs to be confirmed by further experimental research.

## Conclusions

In summary, 28 RNAs that are related to the prognosis of EC patients were identified in this study, and a clinical-mRNA–miRNA–lncRNA prognostic model for EC patients was established. The predictive ability of this clinical-RNA model is significantly better than the clinical-alone model and RNA-alone model in terms of prognosis prediction for EC patients. This study provides a scientific basis for discovering new prognostic markers for EC patients, clarifying the molecular mechanism of EC prognosis, and improving prognosis and clinical management of EC patients.

## Data Availability Statement

The original contributions presented in the study are included in the article/supplementary material, further inquiries can be directed to the corresponding author/s.

## Author Contributions

Conceptualization: XZ and XP. Data curation: FD. Formal analysis: FD and JM. Methodology: CQ. Software: FD and CQ. Writing—original draft: FD, JM, CQ, FY, XL, and XZ. Writing—review and editing: XP.

## Conflict of Interest

The authors declare that the research was conducted in the absence of any commercial or financial relationships that could be construed as a potential conflict of interest.
